# Measures of Connectivity and Dorsolateral Prefrontal Cortex Volumes and Depressive Symptoms Following Treatment With Selective Serotonin Reuptake Inhibitors in Adolescents

**DOI:** 10.1001/jamanetworkopen.2023.27331

**Published:** 2023-08-04

**Authors:** Kyung Hwa Lee, Jiyoon Shin, Jung Lee, Jae Hyun Yoo, Jae-Won Kim, David A. Brent

**Affiliations:** 1 Division of Child and Adolescent Psychiatry, Department of Psychiatry, Seoul National University Hospital and Seoul National University College of Medicine, Seoul, Republic of Korea; 2 Integrative Care Hub, Seoul National University Children’s Hospital, Seoul, Republic of Korea; 3 Department of Psychiatry, The Catholic University of Korea, Seoul St Mary’s Hospital, Seoul, Republic of Korea; 4 Department of Psychiatry, University of Pittsburgh School of Medicine, Pittsburgh, Pennsylvania

## Abstract

**Importance:**

Selective serotonin reuptake inhibitors (SSRIs) are considered a first-line pharmacological treatment for adolescent depression with moderate or higher levels of symptom severity. Thus, it is important to understand neurobiological changes related to SSRIs during the course of treatment for adolescents with depression.

**Objective:**

To examine neurobiological changes associated with SSRI treatment in adolescents with major depressive disorder (MDD) by measuring longitudinal changes in volume and resting-state functional connectivity (rsFC) in the dorsolateral prefrontal cortex (DLPFC), a core region of cognitive control.

**Design, Setting, and Participants:**

This cohort study was conducted with an open-label design. Adolescents with MDD and healthy controls were recruited at the Seoul National University Hospital (Seoul, South Korea). Adolescents with MDD were treated with escitalopram for 8 weeks. Data analysis was conducted between April 2021 and February 2022.

**Main Outcomes and Measures:**

Depressive symptoms were assessed using the Children’s Depression Rating Scale–Revised. The outcome measure was defined as the change in Children’s Depression Rating Scale–Revised scores from week 0 (before treatment) to week 8 (after treatment) or upon termination. Participants completed structural and resting-state functional magnetic resonance imaging (rsfMRI) assessments before (week 0) and after (week 8) SSRI treatment. Repeated measures analysis of variance and liner mixed model analyses were used to examine the longitudinal associations of SSRI treatment with DLPFC volume and rsFC between responders who showed at least a 40% decrease in depressive symptoms and nonresponders who did not.

**Results:**

Ninety-five adolescents with MDD and 57 healthy controls were initially recruited. The final analyses of volume included 36 responders (mean [SD] age, 15.0 [1.6] years; 25 girls [69.4%]) and 26 nonresponders (mean [SD] age, 15.3 [1.5] years; 19 girls [73.1%]). Analyses of rsFC included 33 responders (mean [SD] age, 15.2 [1.5] years; 21 girls [63.6%]) and 26 nonresponders (mean [SD] age, 15.3 [1.5] years; 19 girls [73.1%]). The longitudinal associations of SSRI treatment were more evident in responders than in nonresponders. Responders showed significantly increased right DLPFC volume, decreased bilateral DLPFC rsFC with the superior frontal gyri, and decreased left DLPFC rsFC with the ventromedial PFC after treatment compared with before treatment. Furthermore, increased right DLPFC volume was correlated with decreased rsFC between the right DLPFC and superior frontal gyri after SSRI treatment.

**Conclusions and Relevance:**

The preliminary results of this cohort study suggest that the DLPFC volumetric and rsFC changes may serve as potential neurobiological treatment markers that are associated with symptom improvement in adolescents with MDD.

## Key Points

**Question:**

Is antidepressant treatment associated with volumetric and resting-state functional connectivity (rsFC) changes over time in the dorsolateral prefrontal cortex (DLPFC) in adolescents with major depressive disorder (MDD)?

**Findings:**

In this cohort study of 95 adolescents with MDD and 57 healthy control participants, responders with at least 40% depressive symptom improvement showed increased right DLPFC volume, decreased bilateral DLPFC rsFC with the superior frontal gyri, and decreased left DLPFC rsFC with the ventromedial PFC after treatment compared with before treatment.

**Meaning:**

These findings suggest that DLPFC volumetric and rsFC changes after treatment may represent one set of potential neurobiological treatment markers that are associated with depressive symptom improvement in adolescents with MDD.

## Teaser

This cohort study examines neurobiological changes associated with selective serotonin reuptake inhibitor treatment in adolescents in Korea with major depressive disorder by evaluating longitudinal changes in volume and resting-state functional connectivity in the dorsolateral prefrontal cortex.

## Introduction

Antidepressants are widely used to treat depression in adolescents. Selective serotonin reuptake inhibitors (SSRIs) are known to be the most frequently prescribed antidepressants^1^ and are considered a first-line pharmacological treatment for adolescent depression with moderate or higher levels of symptom severity.^2^ SSRI treatment alone or in conjunction with other treatments, such as cognitive behavioral therapy, showed remission rates of approximately 55% and 60%, respectively, in adolescents.^3^ Given the relatively insufficient efficacy of SSRIs, increasing treatment efficacy is one of the central issues in treating adolescents with major depressive disorder (MDD). One possible way to improve treatment efficacy is to understand the neurobiological changes related to SSRIs during the course of treatment for depression. Thus, it is important to examine neurobiological treatment markers that are associated with improving depressive symptoms.

The literature suggests that neurobiological markers associated with the pathophysiology of depression can be targets for depression treatment.^4^ One neurobiological marker of MDD is the altered prefrontal-limbic circuitry (eg, the dorsolateral prefrontal cortex [DLPFC]–amygdala circuitry) that is associated with inadequate cognitive control and emotional dysregulation.^5,6^ Similarly, there are alterations within cortical circuits, such as the executive control network (ECN) and default mode network, which may be associated with deficient cognitive control over self-referential processing.^7^ A meta-analysis^7^ reported hyperconnectivity between the ECN and default mode network during rest in adults with depression. The ECN, which is involved in cognitive control and the top-down regulation of self-referential processing, may play a central role in the pathophysiology and treatment of depression. The DLPFC, a key region of the ECN, is an important neural substrate for depression and can be targeted to treat depression.^8,9^ Thus, the DLPFC may serve as a target region associated with neurobiological treatment markers of clinical improvement.

Previous studies have demonstrated that SSRI treatment is associated with structural and functional changes in the DLPFC, in adults with MDD. For example, after a 12-week SSRI treatment, adults with depression showed increases in the DLPFC gray matter volume, which, in turn, were associated with greater depressive symptom improvement.^10^ Furthermore, a few task-based functional magnetic resonance imaging (fMRI) studies^11,12^ have reported that SSRI treatment affected neural activation changes in the DLPFC during cognitive and emotional tasks in adults with MDD. Specifically, remitters showed a greater reduction in DLPFC activation during cognitive tasks than nonremitters.^11^ Resting-state fMRI (rsfMRI) research^13,14^ has also examined the effects of SSRI treatment on regional activity (eg, fractional amplitude of low-frequency fluctuations) in the DLPFC during the resting state. After SSRI treatment, regional DLPFC activity during the resting state increased in responders but not in nonresponders. Overall, these studies provide evidence that the DLPFC is involved in the neurobiological processes associated with SSRI treatment.

Despite such supportive findings, more research is needed to improve our understanding of the role of changes in DLPFC volume and activity as potential biomarkers for SSRI treatment. There are several reasons for further research. First, relatively little is known about neurobiological changes following SSRI treatment in adolescents with MDD. Given that adolescence is a developmental period with great importance of cognitive control and emotion regulation development and the protracted prefrontal development,^15^ research regarding longitudinal DLPFC changes after SSRI treatment in adolescents with MDD may be required. Second, neurobiological changes following SSRI treatment as a function of the treatment outcome (response or remission) remain relatively understudied and require further research with larger sample sizes. Prior research^10,12^ with relatively small sample sizes has limited the assessment of neurobiological changes after SSRI treatment that are associated with clinical improvement (eg, responders vs nonresponders). Third, given that the brain is an interactive system, further research is needed to examine resting-state functional connectivity (rsFC) after SSRI treatment. Previous studies^16,17^ have reported that SSRI treatment affects rsFC of the dorsomedial PFC in adults with MDD. However, few studies have examined rsFC changes in the DLPFC, a region important for cognitive control, after SSRI treatment.

Thus, we aimed to examine longitudinal DLPFC volume and rsFC changes associated with SSRI treatment in adolescents with MDD. Given the possibility of a bidirectional association between structural and functional alterations in depression,^18^ we also explored the association of DLPFC volumetric with DLPFC rsFC changes after SSRI treatment in adolescents with MDD. To achieve these aims, adolescents with MDD underwent structural MRI and rsfMRI assessment before and after an 8-week SSRI treatment and completed a questionnaire assessing depressive symptoms at each visit. We hypothesized that the longitudinal changes following SSRI treatment would be more evident in adolescents with MDD who responded to SSRI than in those who did not. For example, according to previous research on the pathophysiology of depression^7^ and SSRI treatment,^10^ adolescents with MDD who responded to SSRI would show a greater increase in DLPFC volume and a greater reduction in rsFC between the DLPFC and prefrontal regions involved in self-referential processing compared with those who did not. Despite the lack of empirical evidence, we hypothesized that there would be significant associations of the DLPFC volumetric with DLPFC rsFC changes after SSRI treatment in adolescents with MDD.

## Methods

### Participants

Adolescents aged 12 to 17 years were recruited by the Seoul National University Hospital. Adolescents with MDD were included if they had a current diagnosis of MDD according to the *Diagnostic and Statistical Manual of Mental Disorders* (Fifth Edition)^19^ criteria using the Kiddie-Schedule for Affective Disorders and Schizophrenia for School-Age Children–Present and Lifetime Version^20^ administered by trained clinical psychologists. Exclusion criteria are described in eAppendix 1 in Supplement 1, and a flowchart depicting participant enrollment is shown in eFigure 1 in Supplement 1. This study was approved by the institutional review board for human participants at the Seoul National University Hospital. The parents provided written informed consent, and the adolescents provided written assent using forms approved by the Seoul National University Hospital review board. This study also followed the Strengthening the Reporting of Observational Studies in Epidemiology (STROBE) reporting guidelines for cohort studies.

### SSRI Treatment Procedures

All adolescents with MDD were treated with escitalopram in an 8-week, open-label trial.^21^ Escitalopram was initiated at a dose of 5 mg per day for the first week and then increased to 10 mg per day at week 2. The dose was adjusted at weeks 4 and 6. The dosages could be adjusted upward in 5-mg or 10-mg increments and downward in 5-mg decrements, up to a maximum of 30 mg per day until reaching the dose sufficient for achieving the therapeutic effects, as determined by the investigator’s (J.-W.K) assessment of symptoms and adverse effects. The doses were then maintained until week 8. Psychotherapy, including cognitive behavioral therapy, was not allowed during treatment. Pill counts were monitored to measure treatment compliance.^22^ The treatment was discontinued if adolescents with MDD were noncompliant (<60% of pills taken) on 2 consecutive visits.

### Depressive Symptom Assessment

Depressive symptoms were assessed using the Children’s Depression Rating Scale–Revised (CDRS-R), which is used to evaluate depression severity in children and adolescents.^23^ The interrater reliability was excellent in this study (intraclass correlation coefficient between 2 interviewers, 0.90). Adolescents with MDD visited the clinic at weeks 1, 2, 4, 6, and 8 of the SSRI treatment and completed the CDRS-R at each visit. The outcome measure was defined as the change in CDRS-R scores from baseline (week 0) to week 8 or upon termination. Adolescents with MDD who showed at least a 40% decrease in the adjusted CDRS-R total score (ie, CDRS-R total score minus 17, which was the minimum possible total score) were classified as responders, and the remainder were classified as nonresponders.^21^

### Structural MRI and rsfMRI Acquisition

T1-weighted structural MRI (sMRI) and rsfMRI data were collected using a 3-T scanner (Trio; Siemens Healthineers). T1-weighted images were obtained using a T1-weighted, 3-dimensional gradient-echo pulse sequence with magnetization-prepared rapid gradient-echo sequencing. The rsfMRI data were acquired with interleaved T2*-weighted echo planar imaging. Details of sMRI and rsfMRI data acquisition are described in eAppendix 1 in Supplement 1. The rsfMRI scan lasted for approximately 9.7 minutes, and 190 volumes were collected. Participants were instructed to relax, think of nothing, and remain awake with their eyes closed.

### sMRI and rsfMRI Preprocessing

**Figure 1.**
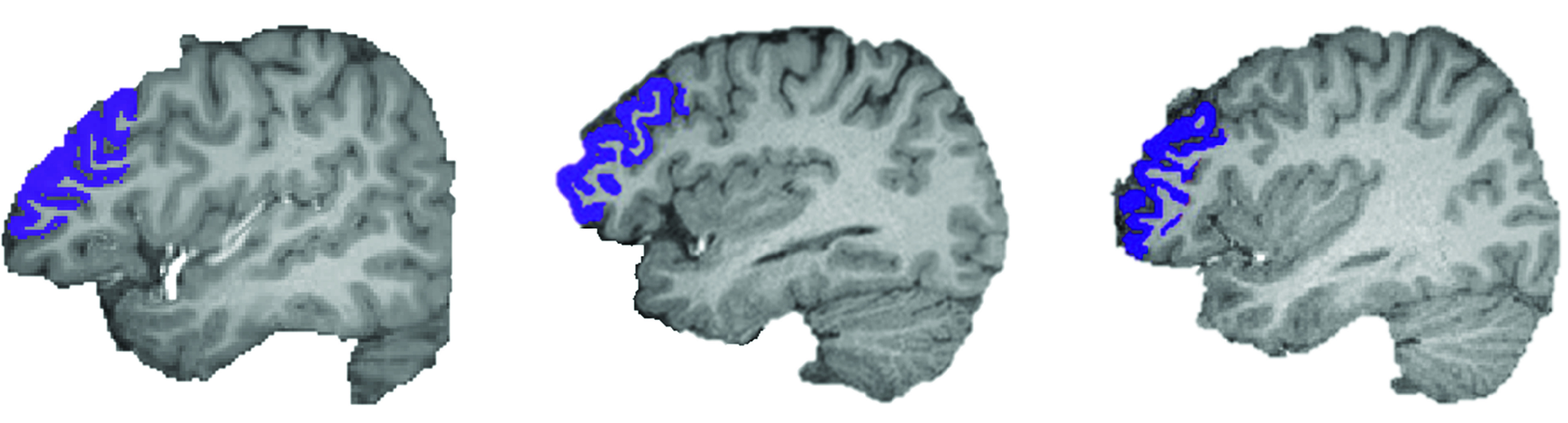
Examples of Dorsolateral Prefrontal Cortex (DLPFC) Seed Regions Segmented by FreeSurfer Images show segmented DLPFC seed regions of 3 separate patients.

T1 and rsfMRI data were preprocessed using FreeSurfer imaging software version 6.0 (Harvard University) and Analysis of Functional NeuroImages imaging software version 21.3.09 (National Institute of Mental Health), respectively. Details of sMRI and rsfMRI data preprocessing and all analytical methods are described in eAppendix 1 in Supplement 1. Briefly, we selected the DLPFC as our seed region defined by the rostral middle frontal gyrus (Figure 1), which is a neuroanatomical representative of and includes core component (Brodmann area 46) of the DLPFC.^24,25^

### Statistical Analysis

We calculated the volumes of the DLPFC seed regions. The extracted DLPFC volumes were exported to SPSS software version 25.0 (SPSS, Inc). We examined whether DLPFC volumes would differ between responders and nonresponders over time (week 0 vs week 8). We also extracted the average time series from the DLPFC seed region and used it to conduct seed-based voxelwise connectivity analyses. Seed-based voxelwise connectivity analyses created each individual’s whole brain correlation map, which contains *z* scores transformed from correlation coefficients (*r* values) using Fisher *r*-to-*z* transformation. We performed Analysis of Functional NeuroImages’ R-based 3dLME function to examine the different longitudinal associations of SSRI treatment with DLPFC rsFC between responders and nonresponders. We also conducted Pearson correlation analyses to examine the associations of DLPFC volume changes with DLPFC rsFC changes before and after SSRI treatment in adolescents with MDD. *P* < .05 (1-sided when calculated with analysis of variance and 2-sided when calculated with *t* tests) was considered statistically significant. Data analysis was conducted between April 2021 and February 2022.

## Results

### Demographic and Clinical Characteristics

A total of 152 adolescents, including 95 adolescents with MDD and 57 healthy controls, were initially recruited (eTable 1 in Supplement 1). After exclusions, 62 adolescents with MDD were included in the volume analysis, and 59 were included in the rsFC analysis. Among the 62 adolescents with MDD included in the volume analysis, 36 (58.1%) were identified as treatment responders (mean [SD] age, 15.0 [1.6] years; 25 girls [69.4%]), and 26 were identified as nonresponders (mean [SD] age, 15.3 [1.5] years; 19 girls [73.1%]). Thirty-three (55.9%) of the 59 adolescents with MDD included in the rsFC analysis were defined as responders (mean [SD] age, 15.2 [1.5] years; 21 girls [63.6%]), and 26 were defined as nonresponders (mean [SD] age, 15.3 [1.5] years; 19 girls [73.1%]). The Table summarizes the demographic and clinical characteristics of the responders and nonresponders. We found a significant difference in the CDRS-R scores at baseline between responders and nonresponders included in the rsFC analysis. No significant differences were found in the mean daily dose of escitalopram over the 8-week treatment period between responders and nonresponders included in the volume or rsFC analyses.

**Table.** Demographic and Clinical Characteristics of Responders and Nonresponders Included in DLPFC Volume and rsFC Analyses Among Participants With Major Depressive Disorder

Variable	DLPFC volume analysis, mean (SD) (n = 62)		Test	*P* value	DLPFC rsFC analysis, mean (SD) (n = 59)		Test	*P* value
	Responders (n = 36)	Nonresponders (n = 26)			Responders (n = 33)	Nonresponders (n = 26)		
Age, y	15.0 (1.6)	15.3 (1.5)	*t* = 0.68	.50	15.2 (1.5)	15.3 (1.5)	*t* = 0.15	.89
Sex, No. (%)								
Female	25 (69.4)	19 (73.1)	*χ^2^* = 0.10^a^	.76	21 (63.6)	19 (73.1)	*χ^2^* = 0.59^a^	.44
Male	11 (30.6)	7 (26.9)			12 (36.4)	7 (26.9)		
Intelligence quotient	103.56 (13.10)	105.58 (16.00)	*t* = 0.55	.59	103.52 (13.55)	105.08 (17.43)	*t* = 0.39	.70
Children’s Depression Rating Scale–Revised score								
Baseline (week 0)	61.47 (11.85)	56.65 (10.72)	*t* = 1.64	.11	61.79 (10.74)	55.81 (10.98)	*t* = 2.10	.04
After treatment (week 8)	35.42 (6.88)	51.38 (11.52)	*t* = 6.30	<.001	35.18 (6.77)	51.00 (11.25)	*t* = 6.33	<.001
Daily dose of escitalopram, mg	13.92 (2.50)	14.42 (2.63)	*t* = 0.76	.45	13.77 (2.56)	14.76 (2.28)	*t* = 1.55	.13
Head motion^b^	NA	NA	NA	NA	0.06 (0.01)	0.06 (0.02)	*t* = 0.99	.33

Abbreviations: DLPFC, dorsolateral prefrontal cortex; NA, not applicable; rsFC, resting-state functional connectivity.

^a^ *χ^2^* for group differences was calculated for categorical variables.

^b^ Head motion was calculated according to the Euclidean distance between the 6 rigid-body head motion parameters for 2 continuous time points.

### Longitudinal Changes (Before vs After Treatment) by SSRI Treatment in Adolescents With MDD

#### DLPFC Volumes

**Figure 2.**
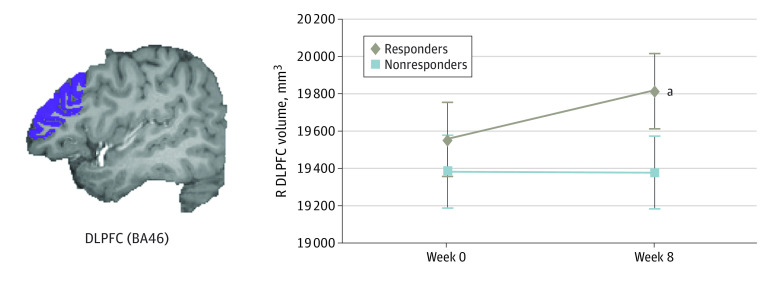
Longitudinal Associations of Selective Serotonin Reuptake Inhibitor (SSRI) Treatment With Right Dorsolateral Prefrontal Cortex (R DLPFC) Gray Matter Volume Gray matter volume in the R DLPFC (left panel, purple shading) increased after SSRI treatment in responders, but not in nonresponders. Error bars in right panel represent 99% CIs. BA46 indicates Brodmann area 46. ^a^*P* = .04.

We found a significant group (responders vs nonresponders) by time (week 0 vs week 8) interaction in the right DLPFC volume, controlling for intracranial volume (*F*_1,59_ = 6.36; *P* = .01; *η_p_*^2^ = 0.097). An increased right DLPFC volume after SSRI treatment compared with before treatment was observed in responders (mean change, 258.29 mm^3^; 99% CI, 70.66 to 445.01 mm^3^; *P* = .04), but not in nonresponders (mean change, −2.02 mm^3^; 99% CI, −210.66 to 207.89 mm^3^; *P* = .87) (Figure 2 and eTable 2 in Supplement 1). However, the left DLPFC volume did not demonstrate a significant group by time interaction (*F*_1,59_ = 0.90; *P* = .35; *η_p_*^2^ = 0.02).

#### rsFC: DLPFC Seed-Based Analysis

**Figure 3.**
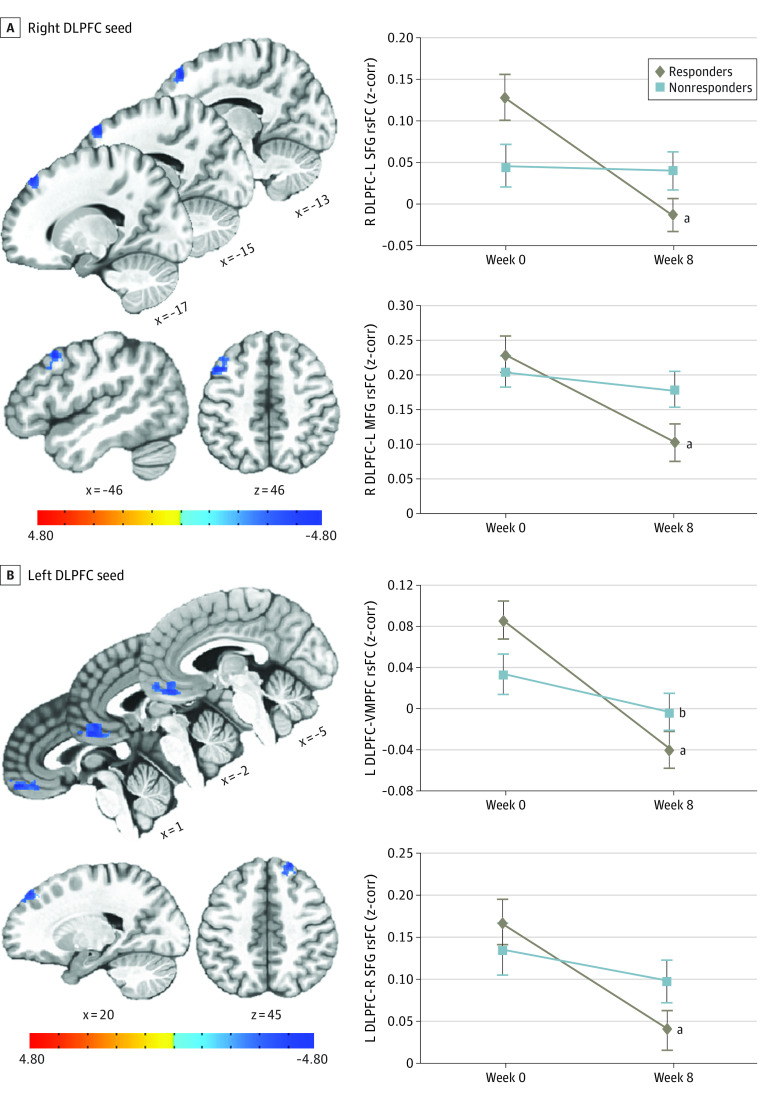
Longitudinal Associations of Selective Serotonin Reuptake Inhibitor (SSRI) Treatment With Dorsolateral Prefrontal Cortex (DLPFC) Resting-State Functional Connectivity (rsFC) A, After SSRI treatment, the rsFCs in the right DLPFC with the left superior frontal gyrus (SFG) (top panel) and left middle frontal gyrus (MFG) (bottom panel) were significantly decreased in responders, but not in nonresponders. B, Similarly, the rsFCs in the left DLPFC with the ventromedial prefrontal cortex (VMPFC) (top panel) and right SFG (bottom panel) were significantly decreased in responders, but less so in nonresponders. Error bars represent standard error of the mean. L indicates left; R, right. ^a^*P* < .01. ^b^*P* < .05.

Group by time interactions were found on the right DLPFC rsFC with the left superior frontal gyrus (SFG) and left middle frontal gyrus (MFG) (eTable 3 in Supplement 1). rsFCs between the right DLPFC and left SFG and between the right DLPFC and left MFG were significantly decreased in responders, but not in nonresponders after SSRI treatment compared with before treatment (Figure 3). Furthermore, there were significant group by time interactions on the left DLPFC rsFC with several brain regions, including the ventromedial prefrontal cortex (VMPFC), right anterior inferior–middle temporal gyrus, right SFG, and left anterior middle–superior temporal gyrus (eTable 3 in Supplement 1). The rsFCs between the left DLPFC and these regions were significantly decreased in responders, but less so in nonresponders, after SSRI treatment compared with before treatment. Figure 3 presents longitudinal changes in rsFCs between the left DLPFC and VMPFC and between the left DLPFC and right SFG. All findings remained significant, even after controlling for baseline depressive symptoms.

### Correlations Between DLPFC Volumetric Changes and DLPFC rsFC Changes After vs Before SSRI Treatment

**Figure 4.**
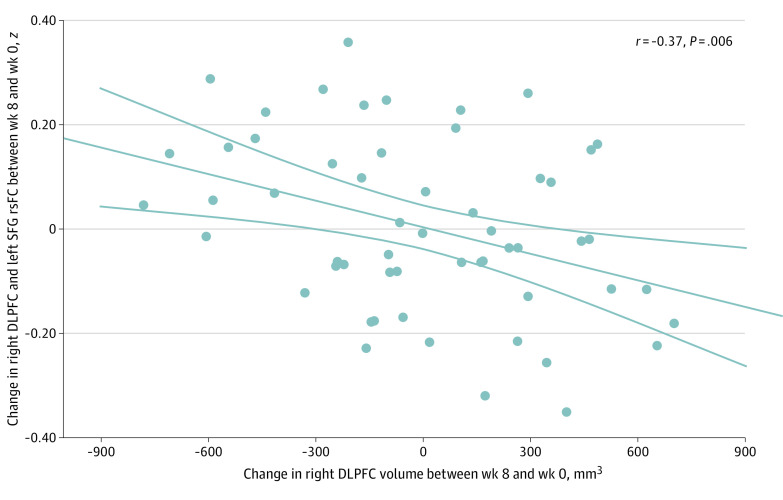
Correlation Between the Right Dorsolateral Prefrontal Cortex (DLPFC) Volume Change and DLPFC–Left Superior Frontal Gyrus (SFG) Resting-State Functional Connectivity (rsFC) Change in Adolescents With Major Depressive Disorder A greater increase in the right DLPFC volume was significantly correlated with a greater reduction in rsFC between the right DLPFC and left SFG.

Given the significant longitudinal associations of SSRI treatment with volume and rsFC in the right DLPFC, we investigated how volumetric changes (week 8 minus week 0) in the right DLPFC were associated with changes in DLPFC rsFC, with 6 regions reported in eTable 3 in Supplement 1, after controlling for intracranial volume. A significant negative correlation was found between the right DLPFC volume change and the right DLPFC–left SFG rsFC change (*r* = −0.37; *P* = .006) in adolescents with MDD. As shown in Figure 4, a greater volumetric increase in the right DLPFC was correlated with a greater decrease in rsFC between the right DLPFC and left SFG. Additional data are shown in eAppendix 2, eFigure 2, eFigure 3, eFigure 4, and eTable 4 in Supplement 1.

## Discussion

To the best of our knowledge, this cohort study is one of the first to investigate neurobiological treatment markers after SSRI treatment that are associated with clinical symptom improvement in adolescents with MDD. We also examined the associations between neurobiological treatment markers, such as DLPFC volume and DLPFC rsFC. First, we found significant longitudinal associations of SSRI treatment with DLPFC volume and rsFC. Compared with nonresponders, responders showed increases in the gray matter volume of the right DLPFC, decreases in DLPFC rsFC with the prefrontal regions involved in cognitive control (eg, the left SFG and MFG), and decreases in rsFC between the left DLPFC and prefrontal regions involved in self-referential processing (eg, the VMPFC). Furthermore, increased right DLPFC volume after treatment was associated with decreased rsFC between the right DLPFC and left SFG.

As hypothesized, the longitudinal associations of SSRI treatment with both DLPFC volume and rsFC were more pronounced in responders than nonresponders. First, the right DLPFC volume increased after SSRI treatment in responders, but not in nonresponders. This result is in line with a previous longitudinal study^10^ that found increased DLPFC volume in adults with MDD after SSRI treatment. The reduced volume of prefrontal regions in patients with depression may be related to neuronal atrophy such as reduced dendritic complexity and spine density.^26^ This atrophy can be reversed using antidepressant drugs.^26^ It is possible that the increased right DLPFC volume after SSRI treatment reflects restored neuronal changes (eg, increased spine density) in the DLPFC with symptom improvement. However, it is not clear whether an 8-week treatment is sufficient for such structural changes; therefore, cautious interpretation is advised.

Second, compared with nonresponders, responders showed significantly reduced rsFC between the right DLPFC and left prefrontal regions (ie, the left SFG and MFG) and between the left DLPFC and right prefrontal regions (ie, the right SFG) over the course of treatment. Elevated rsFCs within the prefrontal regions of cognitive control have been found in adults with MDD.^27^ Reducing elevated rsFC levels have been suggested as a treatment target for depression.^27^ This suggestion is consistent with our findings of reduced rsFC within the prefrontal regions of cognitive control after SSRI treatment, possibly reflecting the decreased use of cognitive resources during resting states. Consequently, this may help adolescents with MDD who responded to SSRI to relax during rest after SSRI treatment. However, this interpretation should be tested in future research by collecting additional information, such as postscan ratings on the thoughts and emotions that the participants had or felt during the resting-state scanning.

Responders also showed greater reductions in rsFC between the left DLPFC and VMPFC, and between the left DLPFC and anterior temporal regions, compared with nonresponders over time. The VMPFC and anterior temporal regions are known to be responsible for self-referential processing. Therefore, reduced rsFC in the left DLPFC with the VMPFC and anterior temporal regions after SSRI treatment may indicate decreased communications between these regions in responders. Hyperconnectivity between the cognitive control regions and self-referential regions of the brain has been shown in depression^7^; therefore, our results indicate that rsFC between the DLPFC and self-referential regions may be normalized in adolescents with MDD who responded to SSRI treatment.

We found some novel results. There was a significant negative correlation between right DLPFC volume change and right DLPFC–left SFG rsFC change after SSRI treatment. Although structural and functional alterations are known to be associated with the pathophysiology and recovery of depression, the association of structural with functional changes in depression remains largely unknown. Structural alterations could lead to altered functional connectivity or vice versa in depression.^18^ Thus, interactive processes may exist between the right DLPFC volumetric change and right DLPFC–left SFG rsFC changes over the course of SSRI treatment, resulting in depressive symptom improvement. However, given that limited information exists about brain structure-function relationships, further research is necessary to elucidate how structural-functional relationships are associated with treatment effects for adolescent depression.

### Limitations

This study has some limitations. First, this study was conducted with an open-label design, which made it difficult to disentangle the effects of SSRI treatment from the effects of nonspecific factors (eg, expectation of improvement or knowledge of treatment). Second, this study treated all adolescents with MDD with 1 SSRI, escitalopram, which further limited the generalizability of our findings across different classes of SSRIs. However, using 1 SSRI for treatment may also have some benefits by excluding possible confounding effects, which may be caused by different SSRIs. Third, given that several factors, including specific symptoms and abuse or trauma history, could alter the effects of SSRI treatment, we did not test the potential moderation because of the lack of available data. Fourth, given the significant baseline difference in right DLPFC–left SFG rsFC between responders and nonresponders, the significant change of this rsFC in responders may be associated with the baseline difference, indicating the potential role of baseline differences in examining longitudinal associations of treatment with rsFC. Fifth, for rsFC analysis, we applied a seed-based approach, which has some advantages, such as a straightforward interpretation of the findings. However, this approach has some limitations, and future research should use a large-scale network analysis approach.^28^

## Conclusions

This study found longitudinal associations of SSRI treatments with both volume and rsFC in the DLPFC of adolescents with MDD using a relatively large sample. More significant longitudinal changes in the DLPFC volume and rsFC in responders may be potential treatment markers of neural plasticity that can mediate the effects of SSRI treatment or be modulated by the beneficial effects of SSRI treatment. Therefore, our findings, while preliminary, underscored the importance of the DLPFC volumetric and rsFC changes as potential neurobiological treatment markers that are associated with symptom improvement in adolescents with MDD.

## Notes

Supplement 1. **eAppendix 1.** Supplemental Methods **eAppendix 2.** Supplemental Results
eReferences
**eTable 1.** Demographic and Clinical Characteristics of Adolescents With MDD and Healthy Controls (HC) at Baseline Included in DLPFC Volume and rsFC Analyses **eTable 2.** DLPFC Volumes (mm^3^) Between Responders, Nonresponders, and Healthy Controls at Baseline (Week 0) and After (Week 8) SSRI Treatment **eTable 3.** DLPFC Seed to Voxel-Wise Connectivity Analysis*: Longitudinal Associations of SSRI Treatment With DLPFC rsFC in Responders and Nonresponders **eTable 4.** Longitudinal Associations of SSRI Treatment With DLPFC rsFC in Responders and Nonresponders: Comparison With HC **eFigure 1.** Flow Diagram of the Participants From Enrollment Through the Analyses **eFigure 2.** Correlations Between the Right DLPFC Volume Change and Depressive Symptom (CDRS-R) Change, and Between the Right DLPFC–Left SFG rsFC Change and Depressive Symptom (CDRS-R) Change in Adolescents With MDD **eFigure 3.** Longitudinal Associations of SSRI Treatment With Right DLPFC Gray Matter Volume: Comparison With HC **eFigure 4.** Longitudinal Associations of SSRI Treatment With DLPFC rsFC: Comparison With HC

Supplement 2.
Data Sharing Statement
