# Supplementary material for: Measures of Connectivity and Dorsolateral Prefrontal Cortex Volumes and Depressive Symptoms Following Treatment With Selective Serotonin Reuptake Inhibitors in Adolescents
Source: JAMA Netw Open. 2023 Aug 4;6(8):e2327331. doi: 10.1001/jamanetworkopen.2023.27331 (PMC10403785; doi:10.1001/jamanetworkopen.2023.27331)
Supplement: Supplement 1. — eAppendix 1. Supplemental Methods eAppendix 2. Supplemental Results eReferences eTable 1. Demographic and Clinical Characteristics of Adolescents With MDD and Healthy Controls (HC) at Baseline Included in DLPFC Volume and rsFC Analyses eTable 2. DLPFC Volumes (mm3) Between Responders, Nonresponders, and Healthy Controls at Baseline (Week 0) and After (Week 8) SSRI Treatment eTable 3. DLPFC Seed to Voxel-Wise Connectivity Analysis*: Longitudinal Associations of SSRI Treatment With DLPFC rsFC in Responders and Nonresponders eTable 4. Longitudinal Associations of SSRI Treatment With DLPFC rsFC in Responders and Nonresponders: Comparison With HC eFigure 1. Flow Diagram of the Participants From Enrollment Through the Analyses eFigure 2. Correlations Between the Right DLPFC Volume Change and Depressive Symptom (CDRS-R) Change, and Between the Right DLPFC–Left SFG rsFC Change and Depressive Symptom (CDRS-R) Change in Adolescents With MDD eFigure 3. Longitudinal Associations of SSRI Treatment With Right DLPFC Gray Matter Volume: Comparison With HC eFigure 4. Longitudinal Associations of SSRI Treatment With DLPFC rsFC: Comparison With HC [file jamanetwopen-e2327331-s001.pdf]

Supplemental Online Content

Lee KH, Shin J, Lee J, Yoo JH, Kim JW, Brent DA. Measures of connectivity and dorsolateral prefrontal cortex volumes and depressive symptoms following treatment with selective serotonin reuptake inhibitors in adolescents. *JAMA Netw Open*. 2023;6(8):e2327331. doi:10.1001/jamanetworkopen.2023.27331

eAppendix 1. Supplemental Methods

eAppendix 2. Supplemental Results

eReferences

eTable 1. Demographic and Clinical Characteristics of Adolescents With MDD and Healthy Controls (HC) at Baseline Included in DLPFC Volume and rsFC Analyses

eTable 2. DLPFC Volumes (mm^3) Between Responders, Nonresponders, and Healthy Controls at Baseline (Week 0) and After (Week 8) SSRI Treatment

eTable 3. DLPFC Seed to Voxel-Wise Connectivity Analysis*: Longitudinal Associations of SSRI Treatment With DLPFC rsFC in Responders and Nonresponders

eTable 4. Longitudinal Associations of SSRI Treatment With DLPFC rsFC in Responders and Nonresponders: Comparison With HC

eFigure 1. Flow Diagram of the Participants From Enrollment Through the Analyses

eFigure 2. Correlations Between the Right DLPFC Volume Change and Depressive Symptom (CDRS-R) Change, and Between the Right DLPFC–Left SFG rsFC Change and Depressive Symptom (CDRS-R) Change in Adolescents With MDD

eFigure 3. Longitudinal Associations of SSRI Treatment With Right DLPFC Gray Matter Volume: Comparison With HC

eFigure 4. Longitudinal Associations of SSRI Treatment With DLPFC rsFC: Comparison With HC

This supplemental material has been provided by the authors to give readers additional information about their work.

© 2023 Lee KH et al. *JAMA Network Open.*

eAppendix 1. Supplemental Methods

Exclusion criteria to limit participation

MDD adolescents were excluded if they had a) any chronic medical diseases, b) a history of psychotic disorders including schizophrenia or bipolar disorder, c) a history of eating disorder, d) any developmental disorders such as autism, e) a history of alcohol or other substance abuse within the past 6 months, f) any neurological or physical diseases, g) first-degree relatives with a history of bipolar I disorder, and h) any psychiatric medications. Adolescents with MDD were medication free/naïve and approximately 80% of them was at their first episode of depression when they participated in this study. Healthy controls (HC) did not have any history of psychiatric illness and were excluded if they had first-degree relatives with any history of psychiatric disorders. Furthermore, all participants were excluded if their intelligence quotient (IQ) was below 70.

Structural MRI (sMRI) and resting-state functional MRI (rsfMRI) acquistion

Our participants underwent MRI sessions to collect their structural MRI (sMRI or T1 image) and rsfMRI data. Both T1-and rsfMRI data were acquired with a 3 T whole-body Tim Trio scanner (Siemens AG) using a 12-channel birdcage head coil. T1 images were obtained using a T1-weighted 3D gradient-echo pulse sequence with magnetization-prepared rapid gradient-echo sequencing (repetition time [TR] = 1900 ms, echo time [TE] = 3.13ms, flip angle = 9°, slice thickness = 0.9 mm, matrix size = 256 × 224 × 176). The rsfMRI data were acquired with interleaved T2*-weighted echo planar imaging (TR = 3000 ms, TE = 40 ms, flip angle = 90°, slice thickness = 4.0 mm, in-plane resolution = 3.4 × 3.4 mm, no gap, 35 axial slices, field of view [FOV] = 240 mm). This rsfMRI scan lasted for approximately 9.7 min, and 190 volumes were collected. Participants were instructed to relax, think of nothing, and remain awake with

© 2023 Lee KH et al. *JAMA Network Open.*

their eyes closed.

sMRI and rsfMRI preprocessing and data analyses

sMRI T1 image. T1 images were visually inspected and excluded if they had artifacts (e.g., head motion) and structural abnormalities (e.g., arachnoid cysts). They were then preprocessed using the longitudinal pipeline of the FreeSurfer 6.0 package (https://surfer.nmr.mgh.harvard.edu/) [1]. First, two T1 images collected at baseline (week 0) and post-treatment (week 8) were processed using the default processing pipeline, called "recon-all' (https://surfer.nmr.mgh.harvard.edu/fswiki/recon-all/). This pipeline includes several processing steps, including motion correction, intensity normalization, Talairach transformation, and skull stripping. The processing steps have been described in a previous study [2]. The quality of the processed T1 images was also inspected. Problems with white matter segmentation were observed near the parietal cortex regions. The problems were corrected using the "control points" (https://surfer.nmr.mgh.harvard.edu/fswiki/FsTutorial/ControlPoints_freeview/). The corrected T1 images were re-run as part of the "recon-all, " and the quality of the reprocessed images was confirmed. Second, an unbiased within-subject 'base' template using two structural T1 images of each participant was created. The two structural T1 images collected at the baseline and post-treatment were aligned to the template, which was an unbiased common space. Third, the longitudinal stream (called 'recon-all –long') was used to process each time point T1 image based on information from the base template. Using such a longitudinal stream to process multiple T1 images collected at different time points allowed us to reduce variability compared to using independent processing (e.g., running the default cross-sectional pipeline multiple times) [1]. The longitudinal procedure began by mapping each T1 image to the base template and followed the same steps as in the cross-sectional default processing pipeline, including intensity

© 2023 Lee KH et al. *JAMA Network Open.*

normalization, Talairach transformation, and so on. During this procedure, the cortical (e.g., dorsolateral prefrontal cortex [DLPFC]) and subcortical regions were automatically parcellated and segmented, and the intracranial volume (ICV) was calculated [3,4]. We selected the DLPFC as our seed region defined by the rostral middle frontal gyrus, which is a neuroanatomical representative and includes core component (Brodmann area 46) of the DLPFC [5,6]. We calculated volumes from the DLPFC seed region. The extracted DLPFC volumes were exported to SPSS software. We examined whether DLPFC volumes would differ between responders (R) and non-responders (NR) over time (week 0 vs. week 8).

rsfMRI data. rsfMRI were preprocessed and then analyzed at the single subject-level and group-level. The preprocessing and analytic procedures are described below.

Preprocessing. fMRI images collected during the 9.7 min resting-state were preprocessed using the afni_proc.py pipeline (https://afni.nimh.nih.gov/pub/dist/doc/program_help/afni_proc.py.html), as implemented in Analysis of Functional Neuroimaging (AFNI) [7]. This pipeline includes several preprocessing steps: despiking, slice-time correction, motion correction, co-registration, normalization, and smoothing. The first four functional volumes were discarded and spikes were removed from the remaining volumes. Spatial alignments, including motion correction, co-registration, and normalization, were performed in three steps. First, the functional volumes were aligned to the base volume, which had a minimum number of voxels with outliers. Second, they were aligned with the structural T1 images. Third, they were warped to the standard space (AFNI's MNI152_T1_2009c) using nonlinear transformation matrices that were calculated after the T1 image was registered to the standard space using nonlinear registration. Finally, the normalized functional volumes were smoothed with a 4-mm full-width half-maximum Gaussian kernel.

© 2023 Lee KH et al. *JAMA Network Open.*

The fast ANATICOR method was used to remove nuisance artifacts from the ventricle and white matters, and artifacts from head motions [8]. To do this, we used ventricle and white matter masks segmented by FreeSurfer and eroded them to avoid potential partial volume effects with gray matter. The time series extracted from the ventricle and white matter masks as well as six rigid-body motion parameters were regressed out. Furthermore, volumes with excessive head motion (greater than 0.2 mm calculated based on the Euclidean distance between the six rigid-body motion parameters) and with outliers (more than 10% of outliers in each volume) were censored to control motion artifacts and outliers. Participants were excluded if they had more than 30% of volumes with excessive head motion, which was defined by the Euclidean distance > 0.2 mm). A bandpass filter (0.01 – 0.1 Hz) was also applied to remove slow fluctuations and high-frequency noises. Finally, after removing such nuisance artifacts, this procedure generated a residual time series that was used to compute correlations (i.e., functional connectivity) among brain regions.

Subject-level and group-level (seed-based voxel-wise) analyses. We extracted the average time series from the bilateral DLPFC ROIs and used it to conduct seed-based voxel-wise connectivity analyses. Seed-based voxel-wise connectivity analyses created each individual's whole brain correlation map, which contains *z*-scores transformed from correlation coefficients (*r* values) using Fisher's r-to-z transformation. We used DLPFC correlation maps to conduct the group-level analyses. A linear mixed-effect model (LME) using AFNI's R-based 3dLME was applied to examine the different longitudinal associations of selective serotonin reuptake inhibitor (SSRI) treatment with DLPFC rsFC between responders and non-responders. The LME included Group (R vs. NR) and Time (week 0 vs. week 8) as fixed-effects to test the main effect of group and time, and their interactions. Individual subjects were included as a random effect. Multiple

© 2023 Lee KH et al. *JAMA Network Open.*

comparisons were corrected using 3dClustSim, with smoothing estimated via AFNI's 3dFWHMx with "acf" procedure (https://afni.nimh,nih,gov, version 20.03.01) [7]. The cluster size was determined based on 10,000 Monte Carlo simulations, second nearest neighbor (NN2) clustering, and a two-sided threshold. Both the cluster-defining threshold ($p < .005$) and cluster size necessary to reach a cluster-wise corrected $p < .025$ ($p <.05/2$ DLPFC ROIs) are reported in the Results section.

Statistical analysis

Demographic characteristics were analyzed using independent-sample *t*-tests for age and intelligence quotient (IQ) and a chi-square test for categorical variables such as sex. Given the significant differences in age and IQ between MDD and HC, any analyses comparing MDD and HC were conducted while controlling for age and IQ. Depressive symptoms assessed by the Children's Depression Rating Scale-Revised (CDRS-R) were compared between adolescents with MDD and HC using analysis of covariance (ANCOVA) while controlling for age and IQ. Longitudinal associations of SSRI treatment with DLPFC volumes were analyzed using repeated measures ANCOVAs with Group (R vs. NR) and Time (week 0 vs. week 8), controlling for age, IQ and ICV. Volumes in the right and left DLPFC were analyzed independently; therefore, we used a Bonferroni correction for multiple tests with a significance level of $p < .025$ ($p <.05/2$ ROIs with right and left DLPFC). Finally, we conducted correlation analyses to examine whether longitudinal changes (week 8 to week 0) in DLPFC volumes and rsFC were associated with longitudinal changes in depressive symptoms (CDRS-R scores), and whether longitudinal changes in DLPFC volume were associated with longitudinal changes in DLPFC rsFC. All statistical analyses were performed using the SPSS software (version 25.0; SPSS Inc., Chicago, IL, USA).

© 2023 Lee KH et al. *JAMA Network Open.*

eAppendix 2. Supplemental Results

Participants included for the DLPFC volume and rsFC analyses

Five participants (2 MDD and 3 HC) withdrew their consent after screening. One participant with MDD was excluded due to depressive symptom improvement after screening. Twelve participants (11 MDD and 1 HC) dropped out before either the pre-treatment or post-treatment MRI assessments. Of the remaining 134 participants, 22 were excluded because of anatomical abnormalities (e.g., arachnoid cyst; 3 MDD and 2 HC) and artifacts (e.g., head motion) (13 MDD and 4 HC). For the volume analysis, four participants (3 MDD and 1 HC) were further excluded owing to technical issues during the application of the longitudinal pipeline for T1 image processing. For the rsFC analysis, 10 participants (6 MDD and 4 HC) were further excluded due to low rsfMRI data quality. Therefore, 62 adolescents with MDD (age mean ± SD = 15.11 ± 1.54, 44 girls) and 46 HC (age mean ± SD = 14.24 ± 1.35, 25 girls) were included for the final DLPFC volume analysis and 59 adolescents with MDD (age mean ± SD = 15.24 ± 1.49, 40 girls) and 43 HC (age mean ± SD = 14.26 ± 1.49, 24 girls) were included for the final rsFC analysis (see eFigure 1 for flow diagram).

As more than 25% of the participants were excluded from the final analyses, we tested demographic differences between participants included and excluded from the DLPFC volume and rsFC analyses. There was only a significant difference in age between the participants included and those excluded from the rsFC analyses ($t(144) = 2.18$, $p = 0.03$, Cohen's $d = 0.40$). The included participants (age mean ± SD = 14.82 ± 1.52) were older than the excluded participants (age mean ± SD = 14.23 ± 1.51). We also compared differences in depressive symptom severity at baseline between MDD participants included and those excluded from the DLPFC volume and rsFC analyses. No significant group differences in depressive symptom

© 2023 Lee KH et al. *JAMA Network Open.*

severity assessed by the CDRS-R was found in either analysis (*p*s > 0.63).

Among the 152 recruited adolescents, 62 adolescents with MDD and 46 HC were included in the final analysis of the sMRI (DLPFC volume) data, and 59 MDD and 43 HC were included in the final analysis of the rsfMRI data (DLPFC rsFC) (for more detailed information, see the Supplement and eFigure 1). All adolescents with MDD included in our final sample completed the 8-week treatment, and therefore had sMRI and rsfMRI data both at baseline and post-treatment. eTable 1 presents the demographic and clinical characteristics of MDD and HC at baseline, including DLPFC volume and rsFC analyses. In both cases of volume and rsFC, adolescents in the MDD group were older and had a lower average IQ than those in the HC group. At baseline, MDD adolescents reported higher scores on the CDRS-R than HC.

Additional analyses

Longitudinal changes in DLPFC rsFC, controlling for demographic information. Despite no significant age and sex differences between responders and non-responders, we examined potential effects of age and sex on results of longitudinal changes in DLPFC rsFC. We found that our findings remained significant after controlling for age and sex (all *p*s < 0.025). Thus, age and sex did not have impact on longitudinal changes in DLPFC rsFC after SSRI treatment.

Longitudinal changes in responders modulated by the left and right DLPFC hemisphere. Given that the left and right DLPFC have different axonal projections to other brain regions [9] and have different rsFC changes after psychotherapy [10], we examined whether changes in DLPFC rsFC in responders after treatment were specific to the left or right DLPFC or general to bilateral DLPFC. We conducted a functional regions of interest (ROIs) analysis based on our longitudinal rsFC findings reported in eTable 3. To conduct this type of analysis, first, we

calculated correlation coefficients (i.e., z values) between the functional ROIs, which were defined from the functional connectivity analysis correlated with either the right DLPFC and left DLPFC seeds, and the opposite DLPFC hemisphere (e.g., z values between the left middle frontal gyrus [MFG] originally correlated with the right DLPFC seed and the left DLPFC seed). Thus, we had correlation coefficients both between the right DLPFC and left MFG and between the left DLPFC and left MFG, resulting in creating the hemisphere factor. This approach allowed us to examine the hemisphere effect by testing Hemisphere x Time interactions in responders.

There were no significant Hemisphere x Time interaction effects in the functional ROIs (all *ps* > 0.07) except the left MFG ($F(1,32) = 5.03$, $p = 0.03$, $\eta_p^2 = 0.136$). Significant main effects of time were found in the 5 functional ROIs (all *p*s < 0.001), indicating that responders showed reduced the DLPFC rsFC after treatment, regardless of the left or right DLPFC. Regarding the MFG functional ROI, the rsFC between the right DLPFC and left MFG (mean rsFC change = 0.13 as presented in Figure 3, bottom left) was greatly decreased after treatment compared to rsFC between the left DLPFC (opposite hemisphere) and left MFG (mean rsFC change = 0.08) in responders. Overall, these findings indicated that longitudinal changes in the right DLPFC-left MFG rsFC (Figure 3, bottom left) may be specific to the right hemisphere, but rsFC changes in other functional ROIs with the DLPFC may be general to bilateral DLPFC in responders. However, this interpretation should be cautious because it is based on the results from only functional ROI analysis.

Correlations with changes in depressive symptoms (CDRS-R scores) and changes in DLPFC volume and rsFC after SSRI treatment compared to pre-treatment. We found significant and stronger longitudinal associations of SSRI with DLPFC volume and rsFC in responders than in non-responders. Given these significant results from comparisons of the

© 2023 Lee KH et al. *JAMA Network Open.*

dichotomized groups (responders vs. non-responders) based on depressive symptom improvement, we further examined whether changes in DLPFC volume and rsFC were significantly correlated with changes in depressive symptoms after SSRI treatment compared with post-treatment (week 8-week 0). There was a significant negative correlation between the right DLPFC volume change and depressive symptom change (r = -0.30, p = 0.02), controlling for ICV. Adolescents with MDD who showed a greater increase in right DLPFC volume after SSRI treatment compared to pre-treatment demonstrated a greater reduction in depressive symptoms (greater symptom improvement) (eFigure 2). Positive correlations were found between DLPFC rsFC changes and depressive symptom change (right DLPFC–left superior frontal gyrus [SFG] rsFC change and depressive symptom change: r = 0.40, p = 0.001; right DLPFC–left MFG rsFC change and depressive symptom change: r = 0.40, p = 0.002; left DLPFC–right anterior temporal gyri rsFC change and depressive symptom change: r = 0.39, p = 0.003; left DLPFC–left anterior temporal gyri rsFC change and depressive symptom change: r = 0.35, p = 0.007). For example, adolescents with MDD who showed a greater reduction in right DLPFC rsFC with the left SFG after SSRI treatment compared to pre-treatment showed a greater reduction in depressive symptoms (greater symptom improvement) (eFigure 2). Overall, the DLPFC rsFC changes (e.g., DLPFC-left SFG RSFC change [r^2 = 0.16]) was more strongly correlated with depressive symptom improvement compared to the DLPFC volumetric change (r^2 = 0.09).

Longitudinal changes in DLPFC volume and rsFC by SSRI: Comparison with HC. We found more significant longitudinal changes in DLPFC volume and rsFC in adolescents with MDD who responded to SSRI than in those who did not. It is possible that such significant longitudinal changes in the DLPFC volume and rsFC in responders may be due to time or the

© 2023 Lee KH et al. *JAMA Network Open.*

course of normal brain development. To exclude this possibility, we compared the longitudinal changes in adolescents with MDD (R vs. NR) with those in HC. We examined differences in longitudinal changes between responders, non-responders, and healthy controls by conducting a Group (R vs. NR vs. HC) x Time (week 0 vs. week 8) interaction effect on the right DLPFC volume, controlling for age, IQ, and ICV. We found a significant Group × Time interaction effect ($F(2, 102) = 8.87$, $p < .001$, $\eta_p^2 = 0.148$). Healthy controls did not show a significant change in the right DLPFC volume over time ($p = 0.95$) (see eTable 2 and eFigure 3). We tested Group (R vs. NR vs. HC) x Time (week 0 vs. week 8) interaction effects on the right and left DLPFC rsFC, controlling for age and IQ. We found a significant Group (R vs. NR vs. HC) x Time (week 0 vs. week 8) interaction effect on the right DLPFC rsFC and left DLPFC rsFC (eTable 4 and eFigure 4). The HC group did not show any significant time effects in any of the rsFCs. These results confirmed that longitudinal changes in DLPFC rsFC after SSRI treatment were not time-dependent.

eReferences

1. Reuter M, Schmansky NJ, Rosas HD, Fischl B. Within-subject template estimation for unbiased longitudinal image analysis. *Neuroimage*. Jul 16 2012;61(4):1402-18. doi:10.1016/j.neuroimage.2012.02.084
2. Fischl B. FreeSurfer. *Neuroimage*. Aug 15 2012;62(2):774-81. doi:10.1016/j.neuroimage.2012.01.021
3. Desikan RS, Segonne F, Fischl B, et al. An automated labeling system for subdividing the human cerebral cortex on MRI scans into gyral based regions of interest. *Neuroimage*. Jul 1 2006;31(3):968-80. doi:10.1016/j.neuroimage.2006.01.021
4. Fischl B, van der Kouwe A, Destrieux C, et al. Automatically parcellating the human cerebral cortex. *Cereb Cortex*. Jan 2004;14(1):11-22. doi:10.1093/cercor/bhg087
5. Phillips JL, Batten LA, Tremblay P, Aldosary F, Blier P. A Prospective, Longitudinal Study of the Effect of Remission on Cortical Thickness and Hippocampal Volume in Patients with

© 2023 Lee KH et al. *JAMA Network Open.*

Treatment-Resistant Depression. *Int J Neuropsychopharmacol*. Mar 30 2015;18(8)doi:10.1093/ijnp/pyv037
6. Zaremba D, Dohm K, Redlich R, et al. Association of Brain Cortical Changes With Relapse in Patients With Major Depressive Disorder. *JAMA Psychiatry*. May 1 2018;75(5):484-492. doi:10.1001/jamapsychiatry.2018.0123
7. Cox RW. AFNI: software for analysis and visualization of functional magnetic resonance neuroimages. *Comput Biomed Res*. Jun 1996;29(3):162-73. doi:10.1006/cbmr.1996.0014
8. Jo HJ, Reynolds RC, Gotts SJ, et al. Fast detection and reduction of local transient artifacts in resting-state fMRI. *Comput Biol Med*. May 2020;120:103742. doi:10.1016/j.compbiomed.2020.103742
9. Petrides M. Lateral prefrontal cortex: architectonic and functional organization. *Philos Trans R Soc Lond B Biol Sci*. Apr 29 2005;360(1456):781-95. doi:10.1098/rstb.2005.1631
10. Taren AA, Gianaros PJ, Greco CM, et al. Mindfulness Meditation Training and Executive Control Network Resting State Functional Connectivity: A Randomized Controlled Trial. *Psychosom Med*. Jul/Aug 2017;79(6):674-683. doi:10.1097/PSY.0000000000000466

© 2023 Lee KH et al. *JAMA Network Open.*

eTable 1. Demographic and Clinical Characteristics of Adolescents With MDD and Healthy Controls (HC) at Baseline Included in DLPFC Volume and rsFC Analyses

	N (%) or mean ± SD							
	DLPFC volume analysis Participants, n = 108				DLPFC rsFC analysis Participants, n = 102			
Variable	MDD, n = 62	HC, n = 46	Test	p value	MDD, n = 59	HC, n = 43	Test	p value
Age (years)	15.11 ± 1.54	14.24 ± 1.35	*t* = 3.07	*p* = .003	15.24± 1.49	14.26 ± 1.38	*t* = 3.39	*p* = .001
Female	44 (71.0)	25 (54.3)	χ^2 = 3.16	*p* = .075	40 (67.8)	24 (55.8)	χ^2 = 1.53	*p* = .216
Intelligence (IQ)	104.40 ± 14.29	110.15 ± 10.47	*t* = 2.41	*p* = .018	104.20 ± 15.26	109.93 ± 9.95	*t* = 2.29	*p* = .024
CDRS-R	59.45 ± 11.55	22.74 ± 4.33	*t* = 22.95	*p* < .001	59.15 ± 11.16	22.65 ± 4.32	*t* = 22.88	*p* < .001
Head motion*					0.06 ± 0.01	0.07 ± 0.02	*t* = 1.43	*p* = .160

Note. MDD, major depressive disorder; HC, healthy controls, DLPFC, dorsolateral prefrontal cortex; rsFC, resting-state functional connectivity; CDRS-R, Children's Depression Rating Scale-Revised

* Head motion calculated based on the Euclidean distance between the six rigid-body head motion parameters for two continuous time points.

© 2023 Lee KH et al. *JAMA Network Open.*

eTable 2. DLPFC Volumes (mm^3) Between Responders, Nonresponders, and Healthy Controls at Baseline (Week 0) and After (Week 8) SSRI Treatment

	MDD, n = 62								HC, n = 46			
	Responders, n = 36				Non-responders, n = 26							
	Week 0		Week 8		Week 0		Week 8		Week 0		Week 8	
Regions	Adj. Mean	SEM	Adj. Mean	SEM	Adj. Mean	SEM	Adj. Mean	SEM	Adj. Mean	SEM	Adj. Mean	SEM
Right DLPFC	19560.25	391.96	19818.54	416.37	19385.35	461.22	19383.33	489.95	20323.68	319.86	20174.67	324.65
Right DLPFC (Week 8 – Week 0)	258.29				-2.02				-149.01			
Left DLPFC	19228.41	368.45	19420.31	371.50	19150.00	433.56	19264.38	437.14	19701.03	336.74	19619.49	334.20
Left DLPFC (Week 8 – Week 0)	191.90				114.38				-81.54			

Note. DLPFC, dorsolateral prefrontal cortex; SSRI, selective serotonin reuptake inhibitor; MDD, major depressive disorder; HC, healthy controls; Adj., adjusted for intracranial volume; SEM, standard error of the mean

© 2023 Lee KH et al. *JAMA Network Open.*

eTable 3. DLPFC Seed to Voxel-Wise Connectivity Analysis*: Longitudinal Associations of SSRI Treatment With DLPFC rsFC in Responders and Nonresponders

Seed regions	Connected regions	Coordinate x	y	z	Voxels	Statistics peak *z*	Repeated Measures ANCOVAs** Group x Time interaction	Pairwise comparisons: Paired t-tests
Right DLPFC	Left superior frontal gyrus***	-17	43	50	194	-4.98	$F(1, 56) = 12.48$, $p = 0.001$, $\eta_p^2 = 0.182$	R: $t(32) = 5.45$, $p < .001$, $d = 0.95$<br>NR: $t(25) = 0.004$, $p = .997$
	Left middle frontal gyrus/precentral gyrus***	-48	15	47	191	-5.01	$F(1, 56) = 6.37$, $p = 0.014$, $\eta_p^2 = 0.102$	R: $t(32) = 5.67$, $p < .001$, $d = 0.99$<br>NR: $t(25) = 1.05$, $p = .305$
Left DLPFC	Ventromedial PFC (rectal/orbital gyrus)***	1	56	-20	442	-4.98	$F(1, 56) = 10.17$, $p = 0.002$, $\eta_p^2 = 0.154$	R: $t(32) = 5.90$, $p < .001$, $d = 1.03$<br>NR: $t(25) = 2.25$, $p = .034$
	Right inferior/middle temporal gyrus	55	-4	-32	210	-3.52	$F(1, 56) = 9.65$, $p = 0.003$, $\eta p^2 = 0.147$	R: $t(32) = 5.72$, $p < .001$, $d = 1.00$<br>NR: $t(25) = 1.11$, $p = .279$
	Right superior frontal gyrus extending to superior medial gyrus***	22	47	45	206	-4.41	$F(1, 56) = 5.63$, $p = 0.021$, $\eta_p^2 = 0.091$	R: $t(32) = 4.70$, $p < .001$, $d = 0.82$<br>NR: $t(25) = 1.55$, $p = .135$
	Left middle/superior temporal gyrus	-64	1	-20	187	-4.95	$F(1, 56) = 8.06$, $p = 0.006$, $\eta_p^2 = 0.126$	R: $t(32) = 6.41$, $p < .001$, $d = 1.16$<br>NR: $t(25) = 2.51$, $p = .019$, $d = 1.00$

Note. DLPFC, dorsolateral prefrontal cortex; SSRI, selective serotonin reuptake inhibitor; rsFC, resting-state functional connectivity; R, responders; NR, non-responders; *d*, Cohen's *d* effect size; CDRS-R, Children's Depression Rating Scale-Revised

* Cluster-defining threshold, $p < 0.005$; cluster size > 181 voxels for the right DLPFC seed and > 188 voxels for the left DLPFC seed to achieve a cluster-wise corrected $p < 0.025$

** controlling for baseline depressive symptoms assessed by CDRS-R scores

*** shown in Figure 3

© 2023 Lee KH et al. *JAMA Network Open.*

eTable 4. Longitudinal Associations of SSRI Treatment With DLPFC rsFC in Responders and Nonresponders: Comparison With HC

Seed regions	Connected regions	Coordinate			Voxels	Statistics	Repeated Measures ANCOVAs[*]	Pairwise comparisons:
		x	y	z		peak *z*	Group[**] x Time interaction	Paired t-tests
Right DLPFC	Left superior frontal gyrus[***]	-17	43	50	194	-4.98	$F(2, 97) = 10.48$, $p < 0.001$, $\eta_p^2 = 0.178$	R: $t(32) = 5.45$, $p < .001$, $d = 0.95$<br>NR: $t(25) = 0.004$, $p = .997$<br>HC: $t(42) = 0.68$, $p = 0.501$
	Left middle frontal gyrus/precentral gyrus[***]	-48	15	47	191	-5.01	$F(2, 97) = 6.32$, $p = 0.003$, $\eta_p^2 = 0.115$	R: $t(32) = 5.67$, $p < .001$, $d = 0.99$<br>NR: $t(25) = 1.05$, $p = .305$<br>HC: $t(42) = 0.02$, $p = 0.982$
Left DLPFC	Ventromedial PFC (rectal/orbital gyrus)[***]	1	56	-20	442	-4.98	$F(2, 97) = 12.32$, $p < 0.001$, $\eta_p^2 = 0.203$	R: $t(32) = 5.90$, $p < .001$, $d = 1.03$<br>NR: $t(25) = 2.25$, $p = .034$<br>HC: $t(42) = 0.36$, $p = 0.718$
	Right inferior/middle temporal gyrus	55	-4	-32	210	-3.52	$F(2, 97) = 15.76$, $p < 0.001$, $\eta p^2 = 0.245$	R: $t(32) = 5.72$, $p < .001$, $d = 1.00$<br>NR: $t(25) = 1.11$, $p = .279$<br>HC: $t(42) = 0.76$, $p = 0.451$
	Right superior frontal gyrus extending to superior medial gyrus[***]	22	47	45	206	-4.41	$F(2, 97) = 11.64$, $p < 0.001$, $\eta_p^2 = 0.194$	R: $t(32) = 4.70$, $p < .001$, $d = 0.82$<br>NR: $t(25) = 1.55$, $p = .135$<br>HC: $t(42) = 1.18$, $p = 0.246$
	Left middle/superior temporal gyrus	-64	1	-20	187	-4.95	$F(2, 97) = 10.20$, $p < 0.001$, $\eta p^2 = 0.174$	R: $t(32) = 6.41$, $p < .001$, $d = 1.16$<br>NR: $t(25) = 2.51$, $p = .019$<br>HC: $t(42) = 0.51$, $p = 0.614$

Note. DLPFC, dorsolateral prefrontal cortex; SSRI, selective serotonin reuptake inhibitor; rsFC, resting-state functional connectivity; R, responders; NR, non-responders; HC, healthy controls; *d*, Cohen's *d* effect size

[*] controlling for age and IQ

[**] R vs. NR vs. HC

[***] shown in eFigure 4

© 2023 Lee KH et al. *JAMA Network Open.*

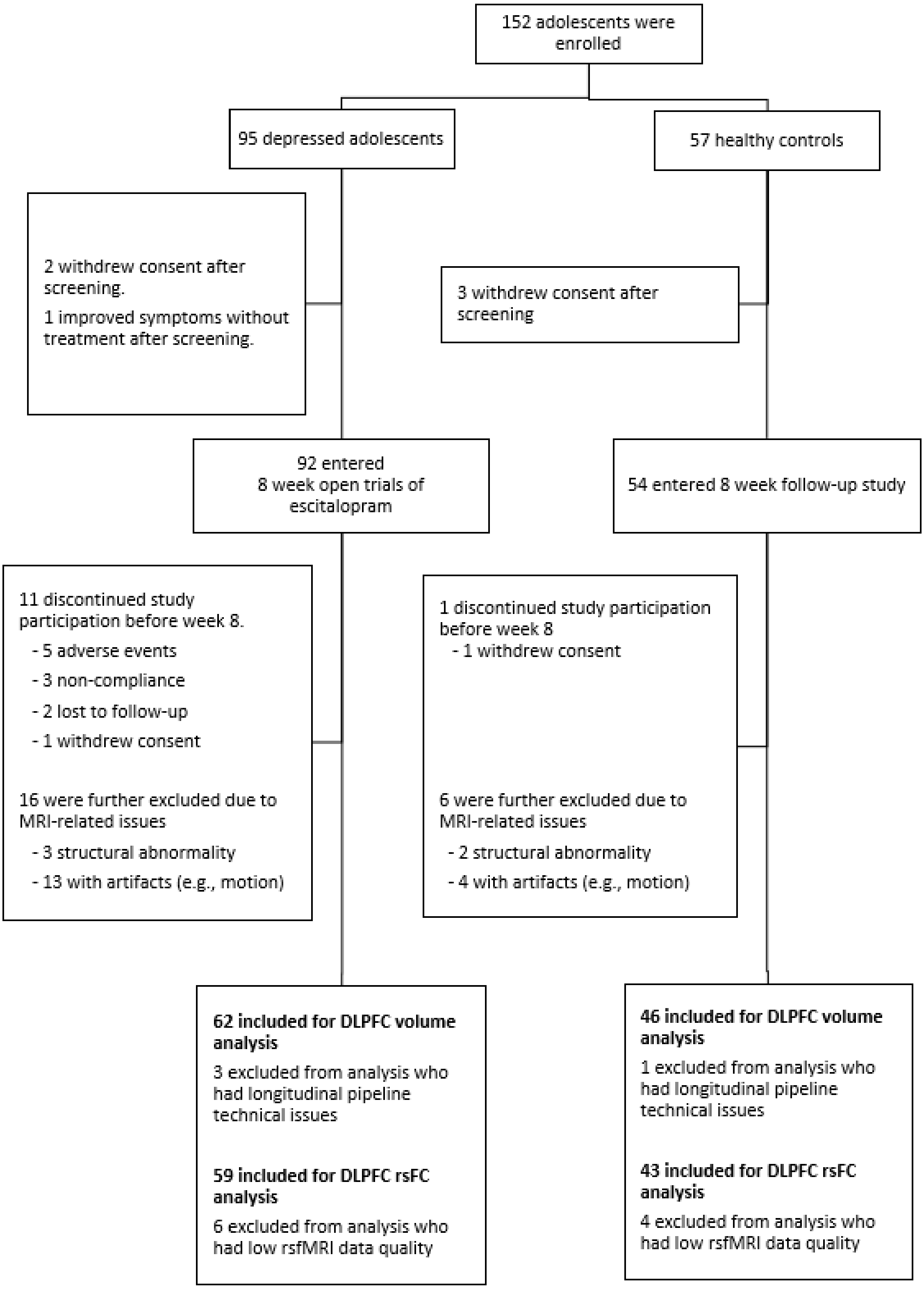

eFigure 1. Flow Diagram of the Participants From Enrollment Through the Analyses
Note. DLPFC, dorsolateral prefrontal cortex; rsFC, resting-state functional connectivity

© 2023 Lee KH et al. *JAMA Network Open.*

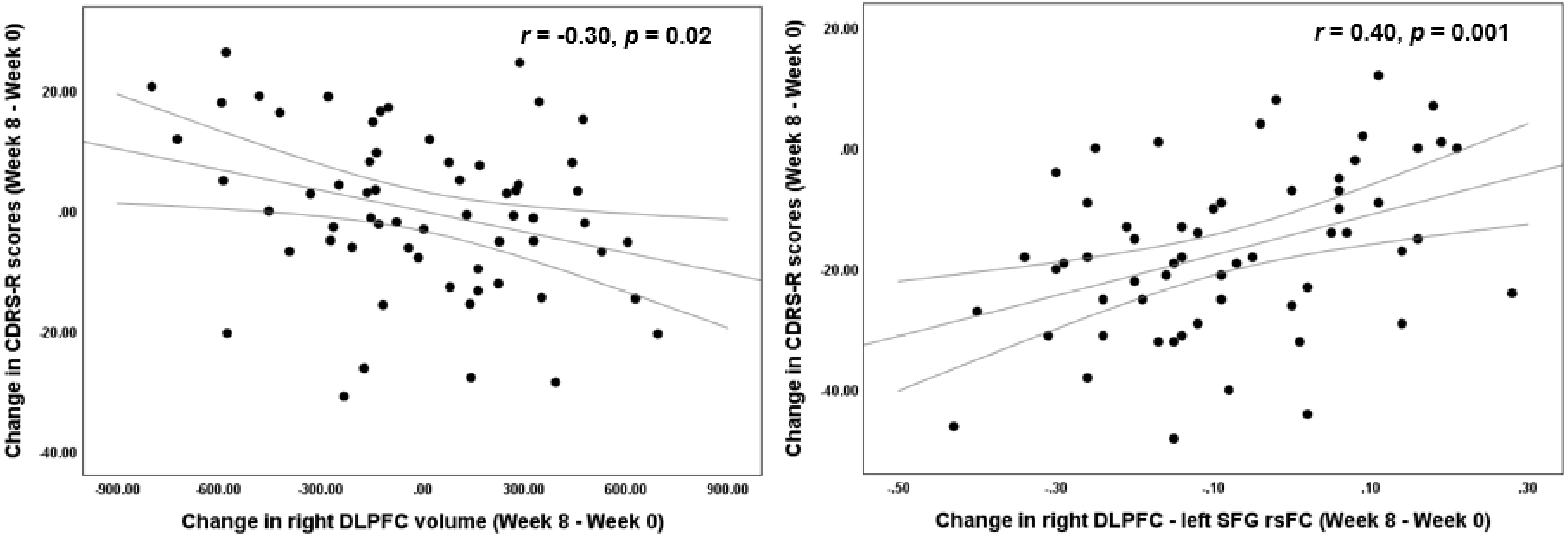

eFigure 2. Correlations Between the Right DLPFC Volume Change and Depressive Symptom (CDRS-R) Change, and Between the Right DLPFC–Left SFG rsFC Change and Depressive Symptom (CDRS-R) Change in Adolescents With MDD. A greater increase in the right DLPFC volume was significantly correlated with a greater reduction (greater improvement) in depressive symptoms. A greater reduction in rsFC between the right DLPFC and left SFG was significantly correlated with a greater reduction (greater improvement) in depressive symptoms.

Note. CDRS-R, Children's Depression Rating Scale-Revised; Week 8, post-treatment; Week 0, baseline/pre-treatment; DLPFC, dorsolateral prefrontal cortex; rsFC, resting-state functional connectivity; SFG, superior frontal gyrus; More negative values in CDRS-R score changes indicate greater reductions (greater symptom improvement) in depressive symptoms.

© 2023 Lee KH et al. *JAMA Network Open.*

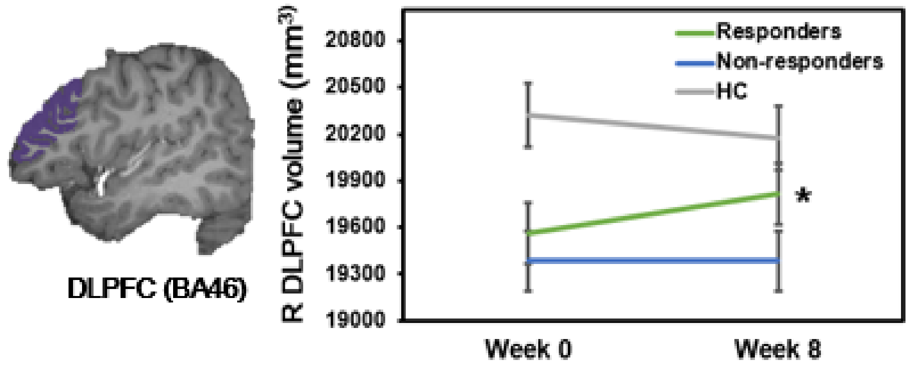

eFigure 3. Longitudinal Associations of SSRI Treatment With Right DLPFC Gray Matter Volume: Comparison With HC. The gray matter volume in the right DLPFC increased after SSRI treatment in responders, but not in non-responders and healthy controls. Error bars represent 99% confidence intervals.
Note. HC, healthy controls; R DLPFC, right dorsolateral prefrontal cortex; BA, Brodmann area; SSRI, selective serotonin reuptake inhibitor
* p <.05

© 2023 Lee KH et al. *JAMA Network Open.*

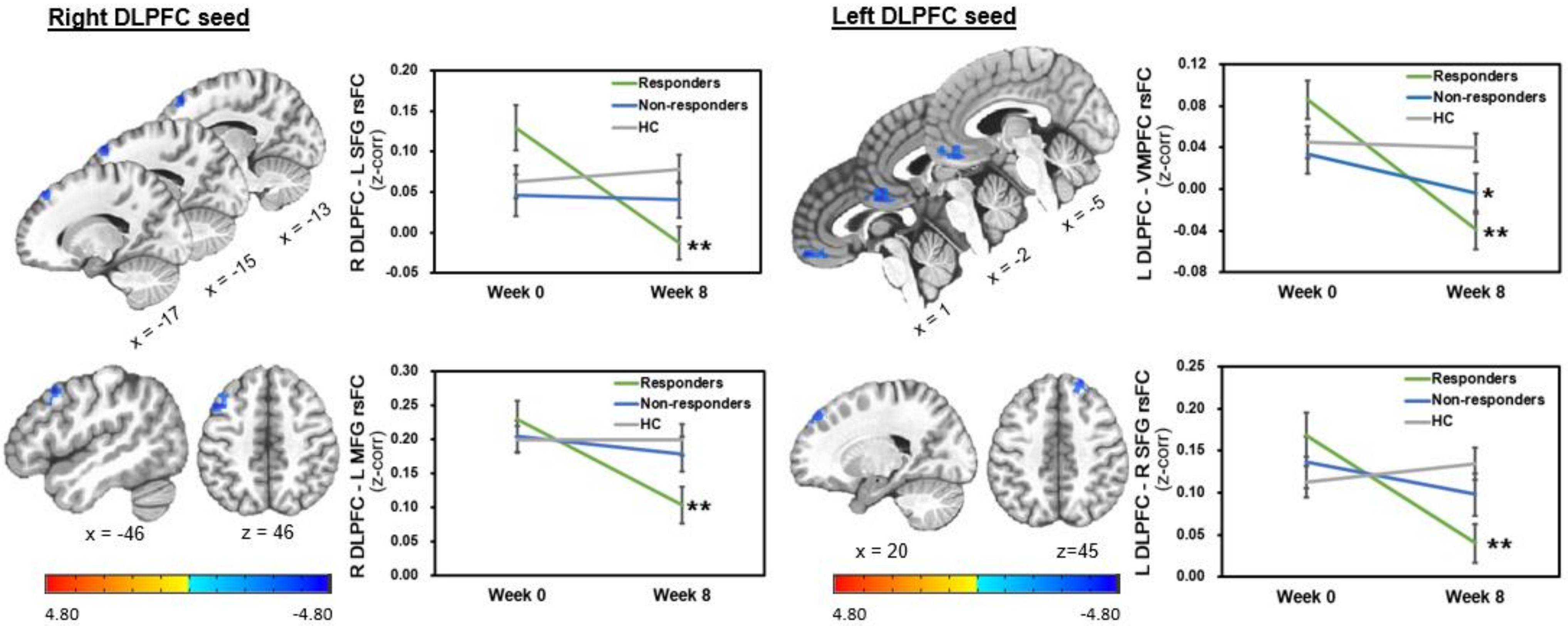

eFigure 4. Longitudinal Associations of SSRI Treatment With DLPFC rsFC: Comparison With HC. The rsFC in the right DLPFC with left SFG and left MFG decreased after SSRI treatment in responders, but not in non-responders and healthy controls. The rsFC between the left DLPFC and VMPFC decreased after SSRI treatment in responders and non-responders, but not in healthy controls. The rsFC between the left DLPFC and right SFG also decreased after SSRI treatment in responders, but not in non-responders and healthy controls. Error bars represent standard error of the mean.

Note. HC, healthy controls; R, right; L, left; DLPFC, dorsolateral prefrontal cortex; rsFC, resting-state functional connectivity; SSRI, selective serotonin reuptake inhibitor; SFG, superior frontal gyrus; MFG, middle frontal gyrus; VMPFC, ventromedial prefrontal cortex.

* *p* <.05

** *p* <.01

© 2023 Lee KH et al. *JAMA Network Open.*
