# Supplementary material for: Measures of Connectivity and Dorsolateral Prefrontal Cortex Volumes and Depressive Symptoms Following Treatment With Selective Serotonin Reuptake Inhibitors in Adolescents
Source: JAMA Netw Open. 2023 Aug 4;6(8):e2327331. doi: 10.1001/jamanetworkopen.2023.27331 (PMC10403785; doi:10.1001/jamanetworkopen.2023.27331)
Supplement: Supplement 2. — Data Sharing Statement [file jamanetwopen-e2327331-s002.pdf]

Data Sharing Statement

Lee. Measures of Connectivity and Dorsolateral Prefrontal Cortex Volumes and Depressive Symptoms Following Treatment With Selective Serotonin Reuptake Inhibitors in Adolescents. *JAMA Netw Open*. Published August 04, 2023. doi:10.1001/jamanetworkopen.2023.27331

Data
Data available: No

Additional Information
Explanation for why data not available: The data that support the findings of this study are available from the corresponding author upon reasonable request.
